# Miniaturized Non-Contact Heating and Transmitted Light Imaging Using an Inexpensive and Modular 3D-Printed Platform for Molecular Diagnostics

**DOI:** 10.3390/s23187718

**Published:** 2023-09-07

**Authors:** Alex Laman, Debayan Das, Aashish Priye

**Affiliations:** 1 Department of Chemical and Environmental Engineering, University of Cincinnati, Cincinnati, OH 45221, USA; lamanac@mail.uc.edu; 2 Chemical Engineering Department, NIT Durgapur, Mahatma Gandhi Rd., A-Zone, Durgapur 713209, West Bengal, India; ddas.che@nitdgp.ac.in; 3 Digital Futures, University of Cincinnati, Cincinnati, OH 45221, USA

**Keywords:** microfluidic heating, miniaturized heater, 3D printed microscope, open source, temperature control, DIY biology

## Abstract

The ability to simultaneously heat and image samples using transmitted light is crucial for several biological applications. However, existing techniques such as heated stage microscopes, thermal cyclers equipped with imaging capabilities, or non-contact heating systems are often bulky, expensive, and complex. This work presents the development and characterization of a Miniaturized Optically-clear Thermal Enclosure (MOTE) system—an open-source, inexpensive, and low-powered modular system—capable of convectively heating samples while simultaneously imaging them with transmitted light. We develop and validate a computational fluid dynamics (CFD) model to design and optimize the heating chamber. The model simulates velocity and temperature profiles within the heating chamber for various chamber materials and sizes. The computational model yielded an optimal chamber dimension capable of achieving a stable temperature ranging from ambient to 95 °C with a spatial discrepancy of less than 1.5 °C, utilizing less than 8.5 W of power. The dual-functionality of the MOTE system, enabling synchronous heating and transmitted light imaging, was demonstrated through the successful execution of paper-based LAMP reactions to detect λ DNA samples in real-time down to 10 copies/µL of the target concentration. The MOTE system offers a promising and flexible platform for various applications, from molecular diagnostics to biochemical analyses, cell biology, genomics, and education.

## 1. Introduction

The past few decades have seen immense growth in the field of microfluidics [1], particularly with the emergence of lab-on-a-chip (LOC) devices that offer profound capabilities for bio-analytical assays, diagnostics, drug discovery, environmental monitoring, and synthetic biology, among other applications [2,3]. LOC devices integrate several laboratory functions into a single portable platform, significantly reducing sample volumes, faster analysis, high-throughput screening, and cost savings. Despite the advancements and benefits, the microfluidic operations inherent to these devices often necessitate a range of peripheral components such as pumps, heaters, and sensors, leading to an intricate and often bulky overall setup [4]. As such, the complexity and size of the support equipment restrict the portability and widespread use of LOC devices, confining them primarily within laboratory environments.

Heating and imaging are a couple of primary operations routinely employed in microfluidics and molecular biology experiments such as the polymerase chain reaction (PCR) [5], cell culture [6], digital microfluidics [7], and isothermal amplification [8]. Heating in microfluidic systems typically involves direct contact between the chip and the heat sources, such as external Peltier heating units or integrated thin-film heaters. These methods can rapidly heat the test sample but consume high power. Additionally, the contact-based heating elements typically obstruct optical access to the heated side, impeding the implementation of transmitted light image analysis. By illuminating the sample from beneath and allowing light to pass through it, transmitted light imaging can reveal structures and details in thin subjects such as cells, tissue samples, or paper substrates not visible in reflected techniques [9,10].

Nucleic acid amplification-based molecular diagnostic tests are another area that primarily relies on simultaneous heating and imaging. The recent outbreaks of viral and bacterial diseases have underscored the critical need for robust, rapid, and widespread diagnostic tools [11,12,13]. These tests, such as real-time PCR and loop-mediated isothermal amplification (LAMP) [8,14], are pivotal in detecting and quantifying specific DNA or RNA sequences of pathogens, genetic mutations, or any target nucleic acid. The amplification process requires precise temperature control to facilitate thermally actuated enzymatic reactions. Simultaneous imaging during these tests provides real-time feedback on the amplification progress, enabling detection and quantification of the target sequence. Diagnostic industries conventionally use thermal cyclers equipped with a metal heating block whose temperature is regulated by energy-intensive Peltier heaters. Despite only a small volume of fluid being heated, a significant portion of the thermal energy is wasted in heating the high-heat-capacity metal blocks, which are not involved in the reaction process. This inefficiency translates into substantial power consumption, typically ranging from ~150–470 W for PCR protocols and ~30 W for isothermal amplification protocols.

In recent years, advances in microfluidic technologies have paved the way for the development of compact, portable diagnostic devices that can perform nucleic acid amplification tests with integrated heating and imaging capabilities [15,16]. These devices primarily rely on micro-Peltier junctions embedded below the microfluidic channel or chamber to provide localized contact heating, substantially reducing power consumption to ~1–3 W. However, their complex fabrication process and limited adaptability inhibit their utility in portable nucleic acid testing systems. Furthermore, these heaters do not permit transmitted light imaging. Other alternatives include non-contact-based heating setups such as hot air guns and laser irradiation. Hot air guns employ forced convection to heat the sample rapidly but consume considerably high power, more than 100 W.

On the other hand, laser consumes low power (1–20 W) but only provides localized heating [17,18]. Furthermore, the complexity and cost associated with such non-contact heating systems limit their broader adoption and make them suboptimal for use in low-resource settings, educational environments, and smaller laboratories. A table summarizing the advantage, disadvantages, and power consumption of heating methods are provided (Supplementary Material; Table S1).

**Figure 1:**
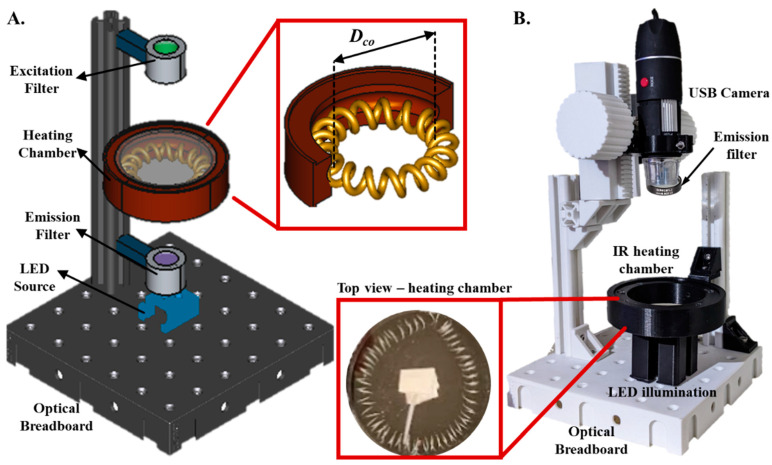
Schematic illustration of the MOTE (Miniaturized Optically-clear Thermal Enclosure) system. (**A**). CAD rendering of the core components and their layout includes an optical breadboard, an LED light source, an emission filter, the heating chamber, and an excitation filter (nonessential components omitted for visual clarity). The Inset figure displays the internal view of the heating chamber with the helically coiled nichrome wire with coil diameter (*D_co_*) less than the chamber diameter. (**B**). An assembled system incorporating all 3D printed components and the digital USB microscope camera (the top acrylic cover of the heating chamber was removed for visual clarity). The Inset figure displays the top view of the heating chamber with the helically coiled nichrome wire with coil diameter, paper substrate, and thermocouple.

Here, we develop and optimize an open-source and 3D-printed Miniaturized Optically-clear Thermal Enclosure (MOTE) system that is transparent from the top and bottom to enable simultaneous heating and transmitted light imaging of microfluidic samples. The heating chamber incorporates a coiled nichrome wire element encased within a cylindrical enclosure (Figure 1A) optimized for effective convective heating while operating on low power. The chamber’s transparency allows for simultaneous heating and image analysis, opening new avenues for real-time monitoring of reactions crucial for numerous diagnostic and research applications. We explore the coupled convective fluid flow and heat transfer dynamics within the chamber to optimize the chamber design parameters for efficient and uniform heating. The resulting miniaturized transparent oven is low-powered (consuming < 10 W) and can reach 95 °C in less than 5 min. We highlight the application of our transparent heating chamber by performing paper-based real-time LAMP reactions, demonstrating the chamber’s ability to simultaneously provide uniform heating and fluorometric imaging for quantification and detection.

## 2. Methods

### 2.1. Design and Engineering of the MOTE System

Our MOTE (Miniaturized Optically-clear Thermal Enclosure) system primarily consists of (i) a light illumination unit, (ii) a heating chamber, and (iii) an imaging unit. The system is modular, with the base consisting of a 6 in × 6 in optical breadboard with threaded screws to assemble the rest of the components. The LED excitation unit is connected directly to the breadboard floor. This unit includes an optical housing with a 3 W RGB LED source and an excitation bandpass filter (SYTO 9 dye excitation—490 nm excitation filter (Thorlabs MDF-TOM, Thorlabs, Newton, NJ, USA). The LED excitation can illuminate the sample from below with different wavelengths and intensities. In the middle section of the setup, the heating chamber was securely attached to a post with adjustable height. The heating chamber comprises a cylindrical enclosure, sealed from the top and bottom with clear acrylic. Within the enclosure, we installed a heating element consisting of helically coiled nichrome wire and a thermal insulating fiber separating the inner walls from the heating element. The coil diameter was 1 cm, while the coil’s outer radius was changed to match the radius of the heated chamber. This configuration allowed for a symmetric heat distribution within the chamber, ensuring uniform heating. The heat generated can be controlled by adjusting the current passing through the wire and considering its resistance per unit length. Ports were cut out to insert a thin-wire K-type thermocouple to monitor the temperature in real-time. The enclosure was fabricated using PLA, a material chosen for its ease of 3D printing. While PLA has a glass transition temperature of ~60 °C and a melting point range of ~220 °C, our design counteracts potential thermal issues by incorporating a thermal insulation layer of 0.5 in thick Ceramic Fiber insulator (McMaster Carr, cat # 93315K82) to separate the nichrome heating element from the enclosure’s walls. In practical operation, as the nichrome wire reaches temperatures of up to 300 °C (representing the upper bound of operational temperature), our experimental data shows that the maximum recorded temperature of the enclosure is 49.4 °C, well below the glass transition temperature of PLA. While PLA has been used in our implementation, materials with higher softening/melting points, such as ABS, could be employed to provide even greater thermal resilience if desired. At the top of the setup, we integrated an imaging system consisting of an optical unit housing emission filter (SYTO 9 dye—520 nm emission filter (Thorlabs MDF-TOM)) and a digital USB microscope (Skybasic, cat # GNIMB401KH03). The camera unit was mounted on a 3D-printed translatable stage that permitted precise z-positioning for optimal imaging.

All custom parts were designed in FreeCAD (version 19) to guide the fabrication process (Figure 1A). The final components were 3D printed using black and white PLA filaments using Prusa MK3s 3D printer (Prusa Research, Prague, Czech Republic). The use of 3D printed components facilitated precise alignment and easy customization of the system according to the specific experimental requirements. It ensured that the system could be adapted for other application-specific modular units. The Arduino Uno R3 microcontroller controlled all the electronics. The power delivered to the nichrome wire, which effectively determined the amount of heat produced, was regulated by an NPN transistor (BD139). This transistor was manipulated using a pulse-width modulation (PWM) signal from a digital pin of Arduino, providing precise control over the current flowing through the wire. A K-type thermocouple, interfaced with a MAX31855 thermocouple amplifier, relayed the internal temperature data to the Arduino. Our selection of a K-type thermocouple was based on several practical considerations. K-type thermocouples are widely used, inexpensive (<$10 U.S.), and easily integrable with open-source microcontrollers such as Arduino. They offer a reliable and accurate means of temperature measurement, with a typical accuracy of ±0.75% and a wide temperature range of −200 °C to ~1200 °C, well beyond the operational range of our microfluidic enclosure. The Arduino dynamically adjusted the power to the nichrome wire using a PID controller to maintain the desired temperature.

The tuning of the PID controller was achieved using the Ziegler-Nichols method. We set integral and derivative gains to zero. We gradually increased the proportional gain until the control system oscillated consistently, marking this as the critical gain (Kc) and noting the oscillation period (P_c_). Using these measurements, we calculated optimal PID values (K_p_ = 0.72, K_i_ = 1.44, K_d_ = 0.09). Operational control of the LED light source and the USB digital camera was managed through momentary push buttons interfaced with the Arduino. We employed a commercially available USB-powered digital camera (Skybasic, cat # GNIMB401KH03) as our imaging sensor, selected for its diverse capabilities, which encompass a manually adjustable focus range from 1 mm to 90 mm, up to 30 fps frame rate, a 40× to 1000× variable magnification ratio, 1920 × 1080 pixels resolution image capture, wireless data transmission, and its broad compatibility with numerous operating systems. We had taken measures to prevent ambient light interference by constructing the enclosure from black acrylic sheets, which completely shields the internal hardware from the external lighting, like a mini darkroom.

The image analysis algorithm employed to quantify fluorescent bioassay signals’ brightness has been described in our previously published work [19]. We first select a circular region encompassing the bioassay for our image analysis. The image analysis algorithm extracts and post-processed RGB values, subsequently applying a gamma transformation to correct for the inherent nonlinearity of pixel intensity values captured by the sensor. This correction relies on predefined gamma and correction factors. Once transformed, the RGB values are converted to CIE XYZ tristimulus values through a direct linear model. These values undergo normalization to separate measures of color luminance (CIE Y value) and chromaticity (x and y values). The application concludes its operation by calculating luminance discrimination (CIE Y Intensity). This ratio provides a color-independent measure of fluorescence signal strength by comparing the luminance values of samples against the background. This setup provided a user-friendly means of controlling the device’s operations. An electronic circuit diagram illustrates the connections between electronic components (Supplementary Material; Figure S1). The cost of constructing the MOTE system is ~$250 (US$) (Supplementary Material; Table S2).

### 2.2. CFD Modeling

A Computational Fluid Dynamics (CFD) model was developed using COMSOL Multiphysics (version 5.5) to simulate and optimize the heat transfer phenomena within the heating chamber. The model was designed as a three-dimensional geometry, exploiting the symmetry of the actual cylindrical chamber for computational efficiency. A 2D rotational symmetry was utilized, modeling the chamber’s cross-sectional “slice” and rotating it around the axis. In the design stage of our model’s geometry, every aspect was parameterized to facilitate flexibility and adaptability. This approach enabled easy adjustments and modifications to the geometry, allowing us to adapt the model seamlessly to changes in the physical design of the heating chamber. To accurately resolve the heat transfer and fluid dynamics within the chamber, a custom mesh was generated with a finer mesh near the nichrome wire and in areas where we anticipated higher temperature gradients, owing to the significant heat generation and dissipation in these regions. A coarser mesh was used in regions distant from the heat source to improve computational efficiency without sacrificing solution accuracy. Grid sensitivity analysis was conducted to determine the optimal mesh density that balances solution accuracy and computational efficiency. Multiple solutions were obtained for various mesh sizes, and the solution was deemed grid-independent when further refinement resulted in less than a 2% change in the peak temperature and fluid velocity.

The model simultaneously implemented fluid dynamics and heat transfer physics to resolve the chamber’s coupled velocity and temperature profiles. The model used the Navier-Stokes equations to describe the nonisothermal flow of air inside the chamber. These equations were coupled with the energy equation to account for the convective and conductive heat transfer between the heating element and the surrounding gas. The properties of air were calculated using the ideal gas law with temperature-dependent properties. The inclusion of radiation heat transfer considered both surface-to-surface and surface-to-ambient radiation. Boundary conditions comprised no-slip conditions on the chamber walls for fluid dynamics, constant temperature of the ambient air outside the chamber to depict the ambient environment and constant heat flux from the nichrome wire. We treated the air in the chamber as an ideal gas with temperature-dependent properties. We utilized a time-dependent solver with a direct linear system solver to ensure computational efficiency. Solver tolerances were tuned for a balance between computational accuracy and speed. Post-processing within the COMSOL environment facilitated data visualization, allowing the examination of temperature distribution, velocity field, and velocity vectors within the chamber. A description of the model, including the geometry, meshing, physics, and solver parameters, is provided (Supplementary Material; Note S1).

### 2.3. Nucleic Acid Amplification Tests

The nucleic acid amplification experiments for paper-based Loop-Mediated Isothermal Amplification (LAMP) and Polymerase Chain Reaction (PCR) were performed within the custom-engineered heating chamber. For LAMP reactions, we employ a paper-based LAMP assay protocol we previously established to carry out the LAMP on paper substrates [20]. Briefly, the LAMP master mix comprised 1.6 μM each of FIP and BIP primers, 0.2 μM each of F3 and B3 primers, and 0.4 μM each of L.F. and L.B. primers. The mixture also contained 8000 U/mL of Bst DNA polymerase (New England Biolabs, County Road, Ipswich, MA, USA), 10× isothermal amplification buffer, which included 2 mM of MgSO_4_ (New England Biolabs, Cat #), 6 mM MgSO_4_, 1.4 mM of each dNTP, and 50 μM of the SYTO 9 dye. A rectangular paper strip (Whatman 113) measuring 3 cm by 1 cm was used as a substrate. A volume of 10 µL from the prepared mix was dispensed onto the designated reagent area of the paper chip. Subsequently, the chip underwent lyophilization utilizing a Labconco FreeZone 2.5 L benchtop freeze dryer. Once the LAMP master mix had dried on the paper substrate, it was ready for testing. The paper substrate was rehydrated using a 25 µL LAMP sample mixture through the inlet to initiate LAMP reactions. For positive controls, 5 μL of target lambda DNA was added. 5 μL of water was added to the paper substrate for the negative control. The paper strips were then incubated within our MOTE heating chamber at a programmed setpoint temperature of 67 °C for 30 min. The tube-based LAMP reactions were carried out in a benchtop qPCR machine (Chai Open qPCR).

Standard PCR tubes containing the required amplification reagents were employed for PCR assays. Primers (Integrated DNA Technologies, Coralville, IA, USA) were designed to amplify 237 base pair targets from a λ-phage DNA template [21]. Briefly, the 100 µL of PCR mix contained 10 µL of 10× Buffer solution (Novagen, Madison, WI, USA; Cat. no. 71085-3), 4 µL 25 mM MgCl_2_ (Novagen, Cat. no. 71085-3), 10 µL of 2 mM dNTPs each (Novagen, Cat. no. 71085-3), 3 µL of 10 µM forward and reverse primers each, 0.8 µL of KOD polymerase (2.5 units/mL, Novagen, Cat. no. 71085-3), 50 μM of the SYTO82 dye, 1 µL of target DNA λ-phage template DNA (Cat. no. N3011S, New England Biolabs) and 67.8 µL of PCR grade water (HyClone, Logan, UT, USA; Cat. no. SH30538.02). The thermal cycling required for PCR was performed by programming the chamber to repeatedly heat and cool between 95 °C (denaturation) and 55 °C (annealing/extension). A list of primer sequences for LAMP and PCR assays used in this study is provided (Supplementary Material; Note S2).

## 3. Results

**Figure 2:**
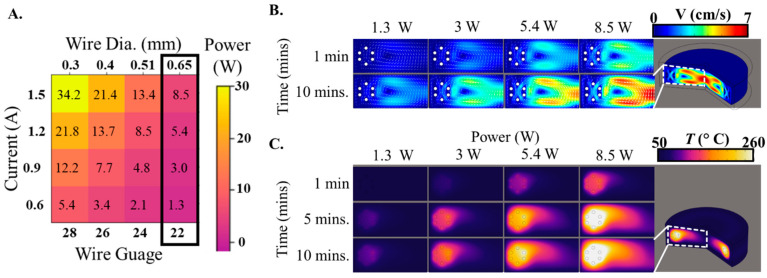
Selecting the heating element and resulting thermal response within the chamber. (**A**). Control power generation via Joule heating by manipulating the helically coiled nichrome wire’s current (I) and resistance (R). The power ranges from 1 W to 35 W. (**B**). Visualization of convection currents driven by thermally induced density gradients, resulting in a circulation pattern within the heating chamber. (**C**). Spatial distribution of temperature gradients near the heating wire and their progressive decrease over time, leading to a near-uniform temperature in the central region of the chamber.

We considered several key factors when designing the heating chamber. The heating element consists of a helically coiled nichrome wire, which can generate varying amounts of Joule heating depending on the current (*I*) passing through the wire and its resistance (*R*) per unit length. Thus, by manipulating these factors, we could control the power generated via Joule heating (*P* = *I*^2^ × *R*), ranging between 1 W and 35 W (Figure 2A). After analyzing different combinations, we decided to use a 22-gauge wire (diameter of 0.65 mm), as it enables the chamber to reach a temperature of up to 100 °C while consuming less than 10 W of power—a significant reduction in comparison to traditional Peltier heating setups. The heat transfer dynamics within the heating chamber encompass all three fundamental modes: convection, conduction, and radiation. Initially, the nichrome wire dissipates heat to the chamber’s internal environment primarily via convection and radiation. The ensuing convection currents then distribute the heat within the chamber, thus elevating the overall chamber temperature. Eventually, the chamber walls, acting as conductors, transfer this heat to the cooler external environment, which is further dissipated through natural or free convection into the surrounding air. Our Computational Fluid Dynamics (CFD) model enabled us to explore the coupling between fluid flow and heat transfer to elucidate the dynamics of convection currents and temperature transients within the heating chamber. The heating element creates thermally induced fluid instabilities known as Rayleigh-Bénard convection in the surrounding cooler air. The thermally induced density gradients between the hot and cold air result in a buoyancy-driven convection current (Figure 2B). The resulting convection currents create a circulation in which air rises near the hot wire, moves towards the center of the chamber, cools down, descends at the center, and then returns to the hot wire along the bottom surface of the chamber. The convection velocity starts relatively small but peaks at 7 cm/s after 10 min at a steady state. At a steady state, the estimated air circulation time within the chamber is approximately 1–3 s. This demonstrates that heat is distributed rapidly throughout the chamber, ensuring a uniform temperature for any sample within the central region. With a similar analysis of the spatial distribution of temperature within the chamber, we observed that most of the temperature gradients are located close to the heating wire (Figure 2C). However, as time progresses, these gradients decrease, suggesting a progressive distribution of heat away from the wire and towards the center of the chamber. After 10 min, the central region of the chamber reaches a near-uniform temperature, with major variations occurring only in the vicinity of the wire.

**Figure 3:**
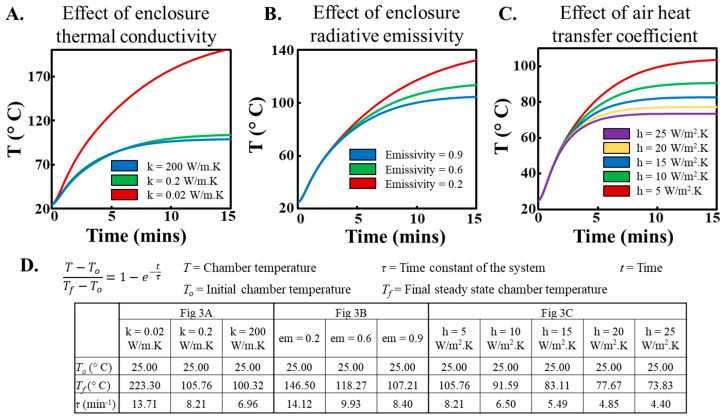
Effect of chamber material properties and ambient conditions on the temperature profiles in the heating chamber (**A**). Influence of the chamber material’s thermal conductivity on the chamber’s temperature profile. Insulating materials, such as foam, effectively trap heat, resulting in higher temperatures that take longer to reach a steady state. (**B**). influence of emissivity of the chamber material on the temperature profile within the chamber. Higher emissivity, exemplified by PLA, facilitates faster thermal equilibrium due to improved heat absorption and radiation. (**C**). Impact of ambient air heat transfer coefficient on the temperature profile within the chamber. (**D**). Table summarizing the temperature and time constants within the chamber modeled as a first-order thermal system for temperature profiles obtained in (**A**–**C**).

To analyze the thermal characteristics, we analyzed the influence of several key system parameters on the resulting thermal response within the chamber. Three materials were chosen to simulate varying levels of thermal conductivity: foam (*k* = 0.02 W/mK; low thermal conductivity), PLA (*k* = 0.2 W/mK; a widely used 3D printing material), and aluminum (*k* = 200 W/mK; high thermal conductivity). Our simulations revealed that highly insulating materials, such as foam, increase the steady-state temperature within the chamber by effectively trapping heat (Figure 3A). However, this comes at the cost of a longer time to reach a steady state. Interestingly, we observed no significant difference between the thermal profiles generated using PLA and aluminum enclosures. Next, we studied the effect of emissivity of the enclosure material, a property that significantly influences the radiation heat transfer. We varied the emissivity to emulate the characteristics of foam (emissivity = 0.6), PLA (emissivity = 0.9), and aluminum (emissivity = 0.2). Our simulations revealed that PLA, with the highest emissivity, reached a steady state the fastest (Figure 3B). This is likely because materials with higher emissivity are better at absorbing and radiating heat; hence, they reach thermal equilibrium faster. Given these benefits and the ease and cost-effectiveness of 3D printing with PLA, we chose it as our preferred enclosure material. External factors were also considered in our model, including the heat transfer coefficient of the ambient air. Heat transfer coefficient values of air can range from 5–25 W/m^2^K and affect the rate at which the chamber loses heat to the surrounding air. Our simulations demonstrated that the chamber’s final steady-state temperature could range from ~65 °C to ~100 °C, corresponding to h = 5 W/m^2^K and h = 25 W/m^2^K, respectively (Figure 3C). The practical implication of this finding is that adding a fan to the system can manipulate the ambient air heat transfer coefficient, enabling a passive manipulation of the chamber’s thermal behavior. The effect of system parameters on the temperature profile can also be analyzed by fitting a first-order response curve to each of the temperature curves in Figure 3. Temperature (T) vs. time (t) data were fit into (T − T_o_)/(T_f_ − T_o_) = (1 − exp(−t/τ)); where τ is the first order response time constant of the system, T_o_ and T_f_ are the initial and final steady-state temperature inside the chamber respectively. We extracted the corresponding time constants (τ), representing the time taken for the temperature to reach 63.2% of the final steady-state value, to serve as a reliable metric to evaluate the heating speed in our system in a quantitative manner (Figure 3D).

**Figure 4:**
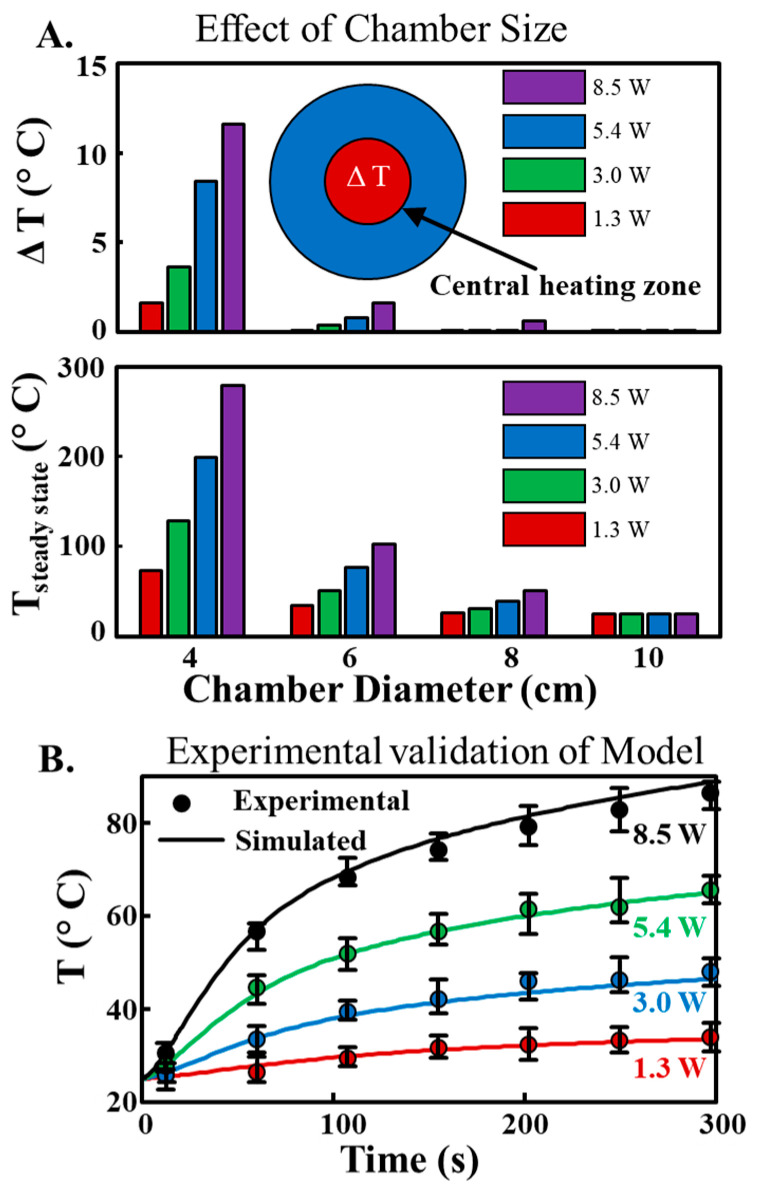
Optimization of Heating Chamber Design and model validation. (**A**). influence of chamber diameter on the spatial temperature variation (top) and steady-state temperatures (bottom) within the central circular region at different output power. The blue circle provides a top representation of the heating chamber. Within this blue circle, the red circle, which is noticeably smaller (diameter = 2 cm), illustrates the designated central circular region where the spatial temperature variation is analyzed. (**B**). comparison of experimentally obtained and simulated temperature profiles for a PLA chamber with a diameter of 6 cm. The close match validates our Computational Fluid Dynamics (CFD) model’s accuracy in predicting the heating chamber’s thermal behavior.

The heating chamber dimension is another important design parameter influencing internal temperature profiles. We simulated transient and steady-state temperature profiles for chambers of different sizes, from 4 cm to 10 cm in diameter. We defined a central circular region of diameter 2 cm in the chamber’s center (red central circle within the chamber as depicted in Figure 4A) to analyze spatial variation of temperature—ΔT. The smaller chamber (Dia. = 4 cm) achieved much higher steady-state temperatures (*T_steady state_*) within the central circular region (up to *T_steady state_* = 270 °C) due to the smaller volume in which the heat was confined. Conversely, larger chambers (Dia. = 10 cm) could only reach a maximum steady-state temperature of 25 °C due to the larger volume and increased surface area for heat loss (Figure 4A). However, smaller chambers exhibited sharper temperature gradients, leading to non-uniform spatial temperature distributions. We quantified this temperature variation (ΔT) by calculating the maximum temperature difference within the central circular region. While smaller chambers offer higher steady-state temperatures, the resulting ΔT values could reach ~12 °C, rendering them unsuitable for most biochemical applications where uniform heating is crucial.

On the other hand, larger chambers provide uniform temperature distribution but cannot provide sufficiently high steady-state temperatures. Therefore, we found a balance between these two extremes with a chamber of diameter 6 cm. This size allows for internal temperatures ranging from ambient to 100 °C (varying the power between 1.3–8.5 W) while maintaining a ΔT value below 1.5 °C, ensuring uniform heating conditions for various biochemical applications. Our CFD model’s validity was confirmed by experiments on a PLA chamber with a diameter of 6 cm. The experimentally obtained temperature profiles closely matched the simulation results, thereby supporting the accuracy of our model in predicting the thermal behavior of the heating chamber (Figure 4B). This comprehensive analysis allows us to optimize the design and operational parameters of the heating chamber, ensuring that it can provide a controlled, uniform thermal environment for a wide range of potential applications.

**Figure 5:**
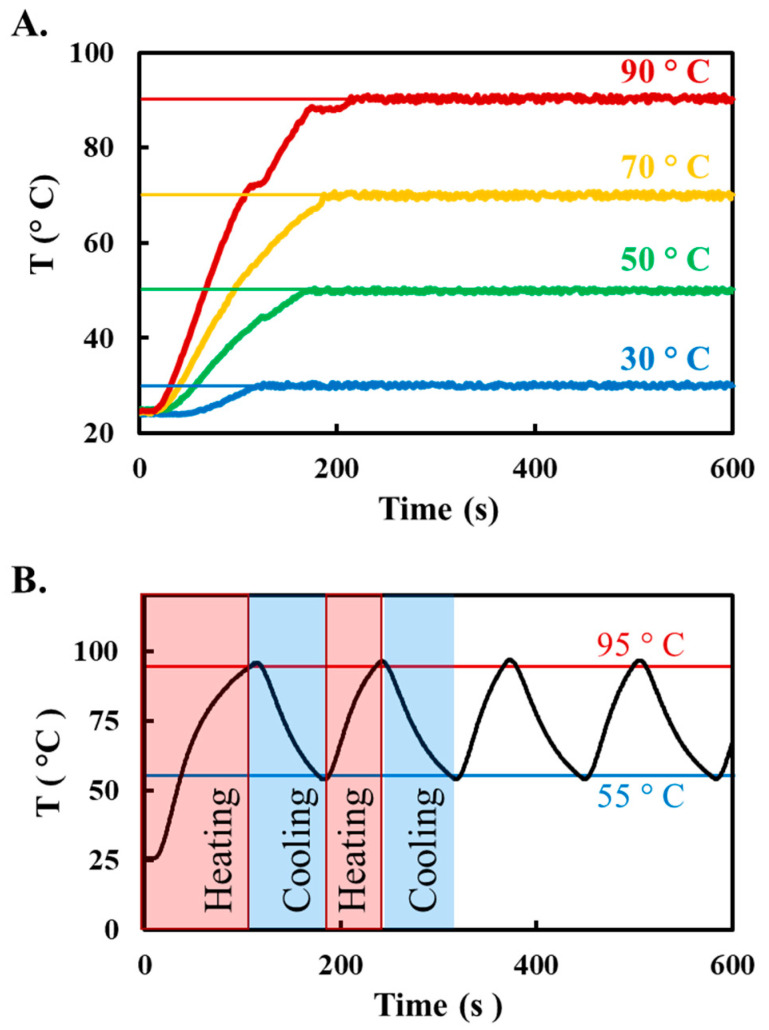
Steady-state temperature profiles and thermal cycling (**A**). Temperature control performance during Isothermal operation. The MOTE system uses PID control to reach and maintain the desired temperature setpoints from 30 °C to 90 °C, with minimum deviation from the setpoint. (**B**). Thermal cycling profile for Polymerase Chain Reaction (PCR) using the MOTE system. The programmed heat-cool cycles mimic the typical thermal cycling parameters for PCR amplification.

To test our MOTE system’s heating capabilities for precise temperature control for nucleic acid testing, we used a heating chamber (chamber diameter = 60 cm), incorporating a 22 gauge nichrome wire as the heating element. We sought to evaluate the utility of our engineered heating chamber in performing steady-state temperature operations and thermal cycling, both of which are widely used in nucleic acid amplification reactions in molecular biology. These reactions have been extensively used in diagnostic applications and often necessitate precise temperature control during their operation. First, we demonstrate steady state operations by implementing a Proportional-Integral-Derivative (PID) controller to regulate the current passed through the nichrome wire and control the generated heat to achieve the desired temperature setpoint in the central circular region inside the chamber. PID constants were selected based on their proficiency in managing system instability and minimizing temperature overshoot. The MOTE system rapidly achieves the predetermined setpoint in under a minute. It sustains a tight temperature regulation, varying no more than ±1.2 °C around the setpoint across a spectrum from 30 °C to 90 °C (Figure 5A).

Furthermore, the MOTE system also exhibits robust capability in performing thermal cycling, a critical requirement for polymerase chain reaction (PCR)—a widely utilized technique in molecular biology. Our system can reliably transition the chamber temperature between high (95 °C) and low (55 °C) thresholds, effectively emulating the cycles of denaturation, annealing, and extension phases of a two-step PCR protocol (Figure 5B). While the temperature cycling rates of the MOTE system may not be as high as those of conventional benchtop thermal cyclers, it accomplishes 30 cycles within an approximate timeframe of 75 min. Despite its slower thermal cycling, the MOTE system can conduct PCR in a simpler, less energy-demanding manner (Supplementary Material; Figure S2), thus underscoring its potential utility in contexts where traditional PCR equipment may be unfeasible or inaccessible.

**Figure 6:**
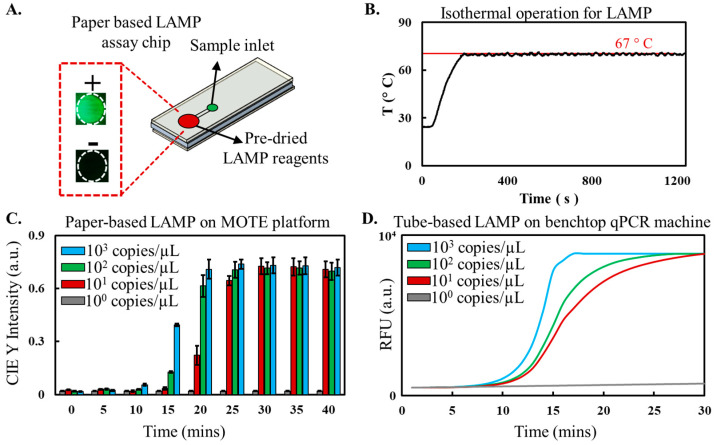
Nucleic acid amplification test using a Paper-LAMP assay on the MOTE system. (**A**). schematic depiction of the paper chip employed in a paper-based LAMP assay to detect λ DNA in the MOTE system. The chip includes stored, dried LAMP reagents and is designed with an inlet to add the target sample (λ DNA). The inset shows the post-amplification fluorescence images of positive and negative samples amplified and imaged (via transmitted light) with the MOTE system. (**B**). Temperature control performance during Loop-Mediated Isothermal Amplification (LAMP) reaction. (**C**). The MOTE platform was used to amplify and image paper-based LAMP chips subjected to a series of samples consisting of λ DNA targets at different concentrations (1–1000 copies/µL) to characterize the assay sensitivity. (**D**). Amplification and detection of traditional tube-based LAMP assays to detect λ DNA using a benchtop q-PCR machine.

We demonstrate the integration of loop-mediated isothermal amplification (LAMP) assay’s resilience with paper microfluidics’ adaptability in our MOTE platform, enabling more accessible in-field nucleic acid testing. Our paper chip design involves a paper substrate with hydrophobic wax barriers printed to direct fluid flow, encased within an acrylic enclosure (Figure 6A). The chip houses an inlet for introducing the target nucleic acid sample and a central region (reaction spot) pre-loaded with freeze-dried LAMP reagents for long-term stability and transportability. Upon sample introduction, the liquid rehydrates the LAMP reagents, and the chip is placed in the MOTE platform’s heating chamber for a 30-minute incubation period to carry out the LAMP reaction. With an internal temperature variation of less than 1.2 °C, the chamber rapidly reaches LAMP-relevant incubation temperatures within 5 min (Figure 6B). Upon completion of the reaction, an LED light source with a SYBR excitation filter illuminates the LAMP assay products from below. The resulting fluorescence is captured by a camera equipped with an SYBR emission filter on top. We convert each pixel’s color information from the RGB color space to CIE-x-y colorimetric space to enhance our ability to quantify the captured fluorescent signals. This transformation process separates color and intensity to produce luminance values with a higher dynamic range [19].

Our experiments confirm the capability of our paper-based LAMP assay chip to reliably detect target λ DNA concentrations as low as ten copies/µL (Figure 6C), indicating its potential utility in scenarios necessitating rapid and efficient nucleic acid testing, particularly in resource-constrained settings. Furthermore, the unique configuration of the MOTE platform, featuring simultaneous heating and transmitted light imaging, facilitates real-time quantification of the paper-based LAMP assay. Per our knowledge, this is the first instance of real-time quantification in a paper-based LAMP assay being reported, a significant advancement enabled solely by the MOTE system’s unique features. Compared to tube-based LAMP assays conducted on a standard q-PCR (Figure 6D), we found a similar sensitivity of 10 copies/µL. This performance parity is significant as it showcases the capability of the paper-based LAMP assays performed in the MOTE platform to offer a comparable sensitivity to the established q-PCR machine while presenting substantial benefits in cost-effectiveness, portability, and ease of use.

## 4. Conclusions

In conclusion, this research presents the design and optimization of a Miniaturized Optically-clear Thermal Enclosure (MOTE) system capable of providing simultaneous temperature control and transmitted light imaging. A CFD model couples the fluid flow and heat transfer inside the miniaturized heating chamber to reveal the effects of key system parameters on the internal velocity distribution, spatial temperature variation, and transient and steady-state temperature profiles within the chamber. The optimal chamber size and material were established by balancing the chamber’s ability to achieve desired temperatures and maintain temperature uniformity. We find that 22-gauge nichrome wire helically coiled around the perimeter of the heating chamber (3D printed from PLA) of diameter 6 cm was optimal for stimulating most thermally actuated biochemical processes. The selected MOTE system enabled the internal temperature to comfortably reach steady state temperatures between room temperature and 95 °C with spatial variation of less than 1.5 °C, all while consuming less than 8.5 W of power. The MOTE system’s capability to concurrently facilitate heating and transmitted light imaging was effectively showcased by conducting paper-based LAMP reactions. This allowed for the real-time detection of λ DNA samples with a sensitivity reaching as low as ten copies/µL of the target concentration.

While the MOTE system demonstrates simultaneous heating and imaging, it does have limitations and scope for improvement. A primary limitation of the MOTE system is the inability to heat and cool samples at rapid rates, thus constraining its potential for rapid PCR. Integrating thicker nichrome wires that can handle increased current would effectively elevate the heating rate within the chamber. Concurrently, introducing an integrated fan outside the chamber would facilitate more efficient cooling. Moreover, material selection for the chamber plays a pivotal role in ensuring its durability under varied thermal conditions. Transitioning to materials like ABS or other high-melting-point polymers would enhance the chamber’s thermal resilience.

Regarding the user interface, there is scope to elevate the MOTE system’s ease of use by replacing the digital microscope with a smartphone camera. This would eliminate the dependency on a computer interface and streamline the post-processing steps. By performing image analysis directly within a smartphone app, the diagnostic process can be more straightforward and broadly accessible, addressing a critical need in point-of-care settings. Furthermore, since the MOTE platform is open source and modular, researchers and developers can contribute to the system’s continual evolution, modifying and enhancing its design and functionality in alignment with emerging research needs. Specifically, customized units could be developed for specific enzymatic reactions or protein folding studies in biochemical analyses. For cell biology studies, modules could be designed to accommodate different cell types or experimental conditions, facilitating in-depth investigations into thermal behavior, heat-shock responses, or the effects of hyperthermia on cancer cells. In educational settings, the MOTE system’s modularity can be adapted to 3D print modules to illustrate heat transfer and microscopy concepts. The MOTE system’s cost-effectiveness and ease of use can also contribute to democratizing laboratory techniques.

## Data Availability

Data is contained within the article or Supplementary Material.

## Supplementary Materials

The following supporting information can be downloaded at: https://www.mdpi.com/article/10.3390/s23187718/s1, Table S1: Comparison of MOTE heating with other heating techniques; Table S2: Part list and cost analysis; Figure S1: Circuit diagram; Figure S2: PCR amplification of λ DNA sample in the MOTE system; Note S1: COMSOL modelling; Note S2: Primer sequences for PCR and LAMP reactions.
