# Supplementary material for: Miniaturized Non-Contact Heating and Transmitted Light Imaging Using an Inexpensive and Modular 3D-Printed Platform for Molecular Diagnostics
Source: Sensors (Basel). 2023 Sep 7;23(18):7718. doi: 10.3390/s23187718 (PMC10535971; doi:10.3390/s23187718)
Supplement: Supplementary file 1 [file sensors-23-07718-s001.zip › sensors-2577654-supplementary.pdf]

Supporting Information for

Miniaturized non-contact heating and transmitted light imaging using an inexpensive and modular 3D-printed platform for molecular diagnostics.

Alex Laman[1], Debayan Das[2] and Aashish Priye[1,3*]

[1] Department of Chemical and Environmental Engineering, University of Cincinnati, Cincinnati, OH 45221, USA

[2] Chemical Engineering Department, NIT Durgapur, Mahatma Gandhi Rd, A-Zone, Durgapur, West Bengal 713209, India

[3] Digital Futures, University of Cincinnati, OH 45221, USA

*Correspondence:

Prof. Aashish Priye Email: piryah@uc.edu

Table of Contents

Table S1: Comparison of MOTE heating with other heating techniques 2

Figure S1: Circuit diagram 3

Table S2: Part list and cost analysis 4

Note S1: COMSOL modelling. 5

1. Model parameters 5
2. Geometry and meshing- 5
3. Heat transfer and fluid flow physics 6

Figure S2: PCR amplification of λ DNA sample in the MOTE system 9

Note S2: Primer sequences for PCR and LAMP reactions. 10

LAMP Primer sequence for amplification of λ phage DNA 10

PCR Primer sequence for amplification of λ phage DNA 10

Table S1: Comparison of MOTE heating with other heating techniques

Heating Method	Advantages	Disadvantages	Estimated Power Consumption
**Traditional Thermal Cycler (Peltier heaters)**	Robust, well-established, High ramp rates	Power-intensive, Expensive, Bulky	~150W – 470W (Thermal cycling),<br>~30W (Isothermal Amplification)
**On-chip micro-Peltier junctions**	Low power consumption	Complex fabrication steps, not readily adaptable to microfluidic chips, contact based heating	~1 – 10 W
**Hot Air Guns**	Rapid heating, Non contact heating	Power-intensive, localized heating	>100 W
**Laser Irradiation**	Non-contact heating, lower power usage,	Expensive, Adds complexity, localized heating	~1-20 W
**MOTE System**	Low power, non-contact heating, inexpensive, Integrated transmitted light imaging	Slow heating ramp rates	1 – 10 W

Figure S1: Circuit diagram

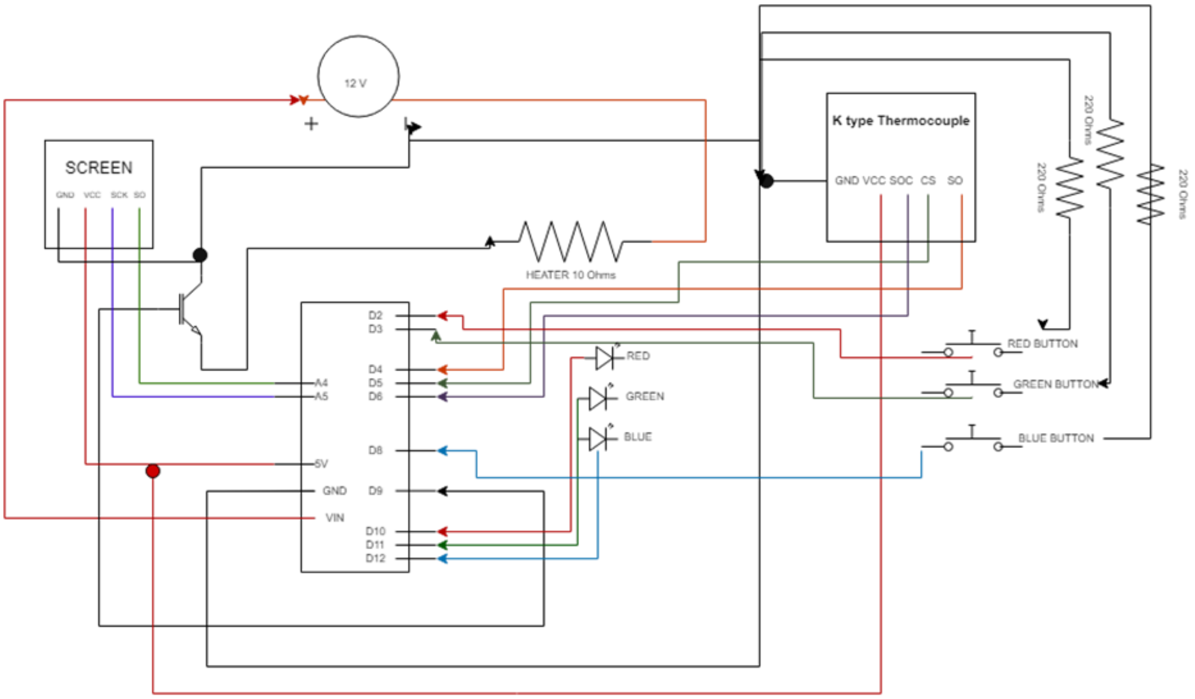

Fig S1: Circuit diagram employed for construction of the MOTE electronics, highlighting all electronic components and their connection to the microcontroller.

Table S2: Part list and cost analysis

Table S2: Parts list and cost for construction of MOTE system. Data are prices at the time of purchase, and may fluctuate based upon the marketplace (eBay, Amazon.com, etc.)

Part Name	Supplier	Quantity	Estimated Cost ($US)
Arduino UNO R3 Microcontroller	Sparkfun	1	20
22 Guage Nichrome wire	Amazon	1	10
100 μF Capacitor	Digikey	1	0.5
K Type Thermocouple	Digikey	1	5
Blue LED	Digikey	1	4
Low-dropout (LDO) Regulator (TLV2217-33)	Digikey	1	1.5
10 μF Capacitor	Digikey	1	0.1
47 μF Capacitor	Digikey	1	0.1
22 pF Capacitor	Digikey	2	0.2 (0.1 each)
Blue Bandpass filter	Thorlabs	1	90
Green Bandpass filter	Thorlabs	1	90
Digital microscope	Skybasic	1	20
**Total ($US)**			**~250**

Note S1: COMSOL modelling.

The COMSOL model simulates the nonisothermal flow of air inside a small cylindrical chamber. The model demonstrates the coupling between energy transport — through conduction, radiation, and convection — and momentum transport induced by density variations in the air.

1. Model parameters

Name	Expression	Value	Description
h0	5[W/(m^2*K)]	5 W/(m²·K)	Heat transfer coefficient
Qf	1.3 [W]	1.3 W	Heat source in filament
p0	50[kPa]	50000 Pa	Initial pressure
rho_enclosure	1185[kg/m^3]	1185 kg/m³	Density, acrylic enclosure
k_enclosure	0.2[W/(m*K)]	0.2 W/(m·K)	Thermal conductivity, acrylic enclosure
Cp_enclosure	1600[J/(kg*K)]	1600 J/(kg·K)	Heat capacity, acrylic enclosure
eps_enclosure	0.94	0.94	Surface emissivity, acrylic enclosure
Mw_a	29[g/mol]	0.029 kg/mol	Molar mass, gas
z2	z3	0.02 m	
z3	z1	0.02 m	
r1	0 [mm]	0 m	
r2	0 [mm]	0 m	
z1	oven_h	0.02 m	
r3	oven_r	0.03 m	
glass_th	3 [mm]	0.003 m	
side_th	3 [mm]	0.003 m	
oven_h	20 [mm]	0.02 m	
oven_r	30 [mm]	0.03 m	
wire_r	0.65[mm]	6.5E−4 m	
wire_pos_r	oven_r - 5 [mm]	0.025 m	
wire_pos_z	2[mm]	0.002 m	
center_pos_r	3 [mm]	0.003 m	r position near the center of the oven
center_pos_z	2 [mm]	0.002 m	z position near the center of the oven
**wire_pos_z**	**2[mm]**	**0.002 m**	
**center_pos_r**	**3 [mm]**	**0.003 m**	**r position near the center of the oven**
**center_pos_z**	**2 [mm]**	**0.002 m**	**z position near the center of the oven**

2. Geometry and meshing-

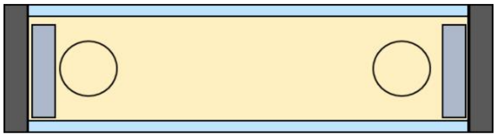

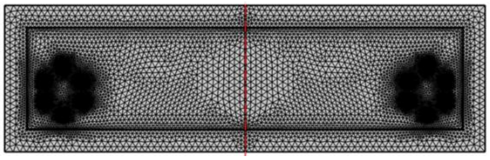

3. Heat transfer and fluid flow physics

Heat transfer in solids and fluids

$$\rho C_p \frac{\partial T}{\partial t} + \rho C_p \mathbf{u} \cdot \nabla T + \nabla \cdot \mathbf{q} = Q$$

$$\mathbf{q} = -k \nabla T$$

In solid domains

$$\rho C_p \frac{\partial T}{\partial t} + \rho C_p \mathbf{u} \cdot \nabla T + \nabla \cdot \mathbf{q} = Q + Q_{\mathrm{ted}}$$

$$\mathbf{q} = -k \nabla T$$

In fluid domains

$$\rho C_p \frac{\partial T}{\partial t} + \rho C_p \mathbf{u} \cdot \nabla T + \nabla \cdot \mathbf{q} = Q + Q_p + Q_{\mathrm{vd}}$$

$$\mathbf{q} = -k \nabla T$$

Thermal insulation

$$-\mathbf{n} \cdot \mathbf{q} = 0$$

Heat source

$$Q = Q_0$$

Heat flux condition

$$-\mathbf{n} \cdot \mathbf{q} = q_0$$

$$q_0 = h(T_{\mathrm{ext}} - T)$$

Momentum transfer equations

$$\rho \frac{\partial \mathbf{u}}{\partial t} + \rho(\mathbf{u} \cdot \nabla)\mathbf{u} = \nabla \cdot [-p\mathbf{I} + \mathbf{K}] + \mathbf{F} + \rho \mathbf{g}$$

$$\frac{\partial \rho}{\partial t} + \nabla \cdot (\rho \mathbf{u}) = 0$$

$$\rho \frac{\partial \mathbf{u}}{\partial t} + \rho(\mathbf{u} \cdot \nabla)\mathbf{u} = \nabla \cdot [-p\mathbf{I} + \mathbf{K}] + \mathbf{F} + \rho \mathbf{g}$$

$$\frac{\partial \rho}{\partial t} + \nabla \cdot (\rho \mathbf{u}) = 0$$

$$\mathbf{K} = \mu\left(\nabla \mathbf{u} + (\nabla \mathbf{u})^T\right) - \frac{2}{3}\mu(\nabla \cdot \mathbf{u})\mathbf{I}$$

Initial Values 1

EQUATIONS

$$p_{\mathrm{init}} = p + p_{\mathrm{hydro}}$$

$$p_{\mathrm{hydro}} = \rho_{\mathrm{ref}}\, \mathbf{g} \cdot (\mathbf{r} - \mathbf{r}_{\mathrm{ref}})$$

Wall 1

EQUATIONS

$\mathbf{u} = \mathbf{0}$

Gravity 1

EQUATIONS

$$\rho\frac{\partial \mathbf{u}}{\partial t} + \rho(\mathbf{u}\cdot\nabla)\mathbf{u} =$$
$$\nabla\cdot[-p\mathbf{I} + \mathbf{K}] + \mathbf{F} + \rho\mathbf{g}$$

Surface-to-Surface Radiation

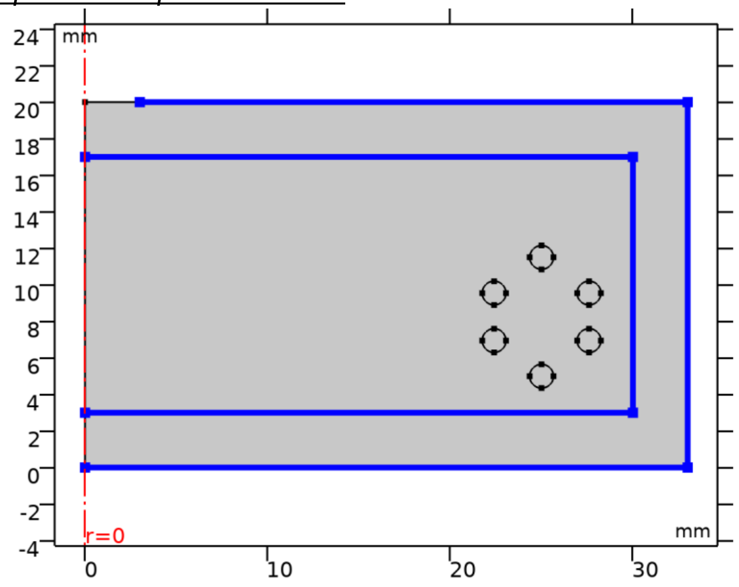

Surface-to-Surface Radiation

EQUATIONS

$$J = \varepsilon e_b(T) + \rho_d G$$
$$G = G_m(J) + G_{amb} + G_{ext}$$
$$G_{amb} = F_{amb} e_b(T_{amb})$$
$$e_b(T) = n^2 \sigma T^4$$

FEATURES

**Diffuse Surface 1**
**Initial Values 1**
**Axial Symmetry 1**

Diffuse Surface 1

EQUATIONS

$$J = \varepsilon e_b(T) + \rho_d G$$
$$\varepsilon + \rho_d = 1$$
$$G = G_m(J) + G_{amb} + G_{ext}$$
$$G_{amb} = F_{amb} \varepsilon_{amb} e_b(T_{amb})$$

$$G_{\text{ext}} = q_s + I_{\text{diff}}$$
$$e_b(T) = n^2 \sigma T^4$$

Nonisothermal Flow 1

EQUATIONS

$$Q_p = \alpha_p T\left(\frac{\partial p_A}{\partial t} + \mathbf{u} \cdot \nabla p_A\right)$$
$$\alpha_p = -\frac{1}{\rho}\left(\frac{\partial \rho}{\partial T}\right)_p$$
$$Q_{\text{vd}} = \tau : \nabla \mathbf{u}$$

Heat Transfer with Surface-to-Surface Radiation 1

EQUATIONS

$$-\mathbf{n} \cdot \mathbf{q} = \varepsilon\left(G - e_b(T)\right)$$

Figure S2: PCR amplification of λ DNA sample in the MOTE system

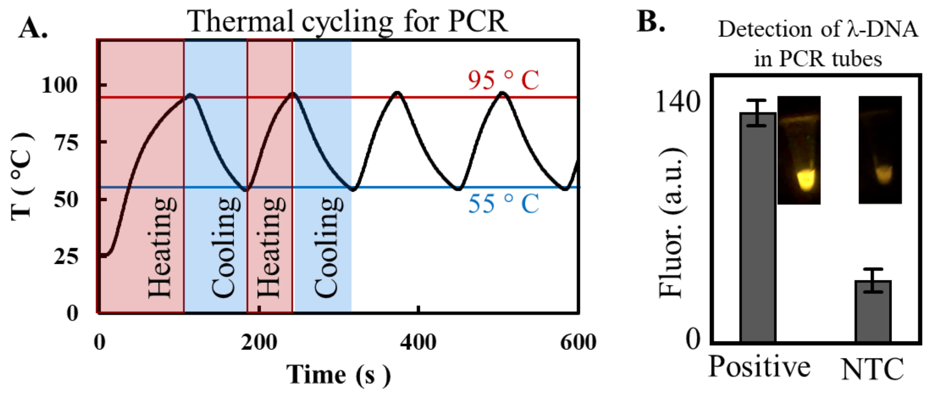

Figure S2: PCR amplification of λ DNA sample in the MOTE system. **A.** Thermal cycling profile for Polymerase Chain Reaction (PCR) using the MOTE system. **B.** Transmitted light fluorescent emission from PCR tubes, indicating successful amplification, captured through the transparent top of the MOTE system during PCR.

Note S2: Primer sequences for PCR and LAMP reactions.

LAMP Primer sequence for amplification of λ phage DNA

Name	Sequence (5`-->3`)	Description	Total length	GC%	TM
**FIP (F1c-F2)**	CAGCATCCCTTTCGGCATACCA-GGTGGCAAGGGTAATGAGG	FIP primer	41	56.1	69.5
**BIP (B1c-B2)**	GGAGGTTGAAGAACTGCGGCAG-TCGATGGCGTTCGTACTC	BIP primer	40	57.5	69.4
**F3**	GAATGCCCGTTCTGCGAG	F3 primer	18	61.1	56.7
**B3**	TTCAGTTCCTGTGCGTCG	B3 primer	18	55.6	55.2
**LF**	GGCGGCAGAGTCATAAAGCA	Loop forward primer	20	55	57.9
**LB**	GGCAGATCTCCAGCCAGGAACTA	Loop backward primer	23	56.5	60.4

PCR Primer sequence for amplification of λ phage DNA

Primers	Annealing sequence (5`-->3`)	Description	GC%	TM
237bp-F	CTGAGGCCGGGTTATTCTTG	Forward primer	55	54.8
237bp-R	CGACTGGCCAAGATTAGAGA	Reverse primer	50	53.1

All primer sequences from order from Integrated DNA Technologies (IDT)
